# MRI of rectal cancer—relevant anatomy and staging key points

**DOI:** 10.1186/s13244-020-00890-7

**Published:** 2020-09-03

**Authors:** Inês Santiago, Nuno Figueiredo, Oriol Parés, Celso Matos

**Affiliations:** 1 Radiology Department, Champalimaud Foundation, Avenida Brasília, 1400-038 Lisbon, Portugal; 2 Nova Medical School, Campo Mártires da Pátria 130, 1169-056 Lisbon, Portugal; 3 Champalimaud Research, Champalimaud Foundation, Avenida Brasília, 1400-038 Lisbon, Portugal; 4 Colorectal Surgery, Digestive Unit, Champalimaud Foundation, Avenida Brasília, 1400-038 Lisbon, Portugal; 5 Radiation Oncology Department, Champalimaud Foundation, Avenida Brasília, 1400-038 Lisbon, Portugal

**Keywords:** Anatomy, Anatomic variants, Rectum, Rectal cancer, Staging, Magnetic resonance

## Abstract

Rectal cancer has the eighth highest cancer incidence worldwide, and it is increasing in young individuals. However, in countries with a high human development index, mortality is decreasing, which may reflect better patient management, imaging being key. We rely on imaging to establish the great majority of clinical tumour features for therapeutic decision-making, namely tumour location, depth of invasion, lymph node involvement, circumferential resection margin status and extramural venous invasion. Despite major improvements in technique resulting in better image quality, and notwithstanding the dissemination of guidelines and examples of standardised reports, rectal cancer staging is still challenging on the day-to-day practice, and we believe there are three reasons. First, the normal posterior pelvic compartment anatomy and variants are not common knowledge to radiologists; second, not all rectal cancers fit in review paper models, namely the very early, the very low and the mucinous; and third, the key clinical tumour features may be tricky to analyse. In this review, we discuss the normal anatomy of the rectum and posterior compartment of the pelvis, systematise all rectal cancer staging key points and elaborate on the particularities of early, low and mucinous tumours. We also include our suggested reporting templates and a discussion of its comparison to the reporting templates provided by ESGAR and SAR.

## Key points

- Imaging is the pillar upon which therapeutic decisions are made in rectal cancer patients.
- Knowledge of normal anatomy and variants of the posterior pelvic compartment is mandatory for rectal cancer staging.
- We provide a roadmap for rectal cancer staging which includes anatomy and anatomic variants of the posterior pelvis, all key staging features and the particularities of early, low and mucinous tumours.

## Background

Rectal cancer is one of the best examples of success of clinical research in the past 40 years. Total mesorectal excision (TME) alone, as opposed to blunt “pelvic rape”, resulted in an increase in the 5-year cancer-specific survival rate from 38 to 68% [1]. The introduction of preoperative chemoradiation therapy in high-risk patients reduced local recurrence rates to 6% as opposed to 13% when given post-operatively [2]. We have slowly moved from specialty-centred decisions to generalised multidisciplinary patient management in which radiology became the pillar for risk stratification. In fact, with a differentiated multimodality treatment based on dedicated preoperative MR imaging, local recurrence has lost relevance compared to early diagnosis, treatment-related morbidity and metastatic disease prevention and control [3]. Despite major improvements in technique resulting in better image quality, and notwithstanding the dissemination of guidelines and examples of staging templates, rectal cancer staging is still challenging. It requires a thorough convolution of radiologists with normal anatomy and technical pitfalls and a clear and systematised knowledge of the key imaging features of rectal cancer for decision-making. In this review, we have first focused on the normal anatomy of the posterior compartment of the pelvis, including the definitions of high/mid/low rectum, compartment boundaries, rectal blood supply and lymphatic drainage, and then systematised the staging key points, namely T staging and sub-staging, N staging and tumour deposits, M staging, circumferential margin of resection and extramural venous invasion. We have further included a discussion on the particularities of early, low and mucinous rectal cancers and provided our own staging templates. Even though no paper will ever replace validated experience and advice from senior experts, we aim to contribute to an easier and smoother training of interested radiologists.

## Main Text

### MR imaging of the normal rectum

#### From where up to where down

**Fig. 1:**
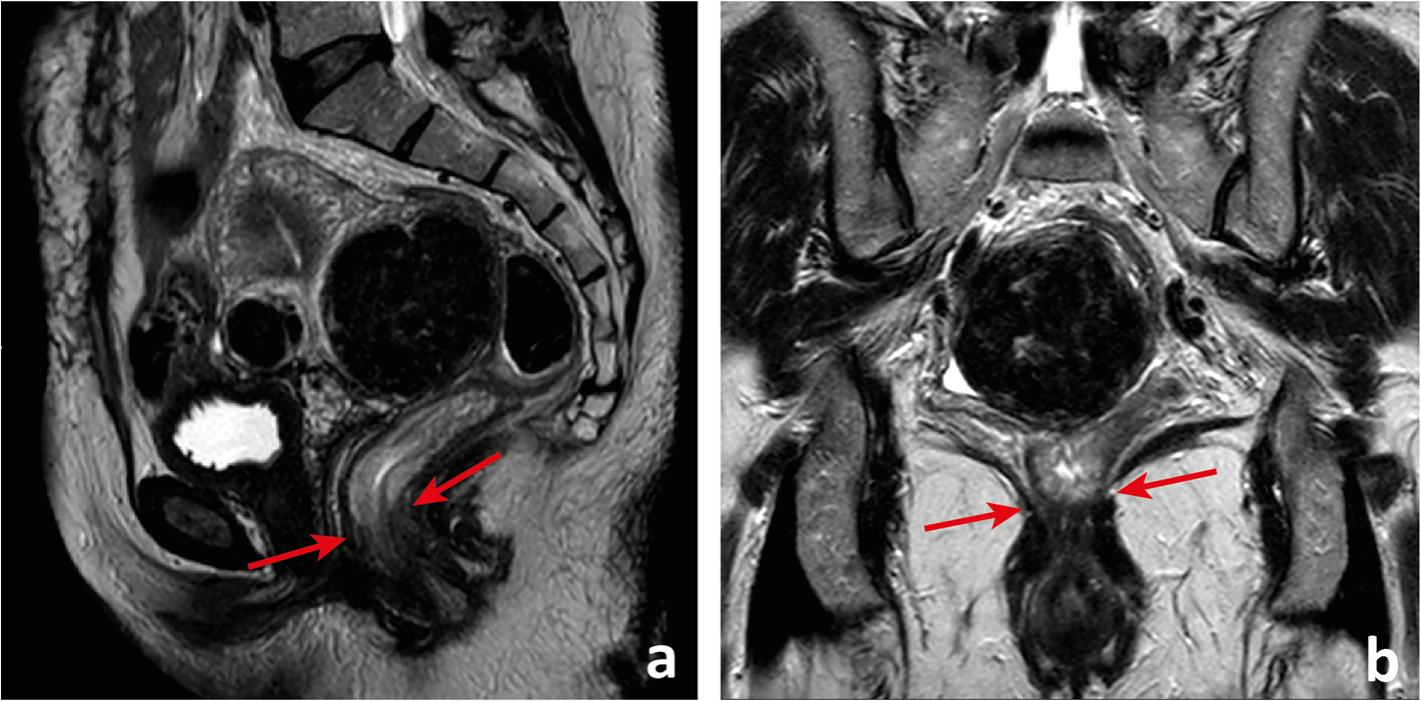
The anorectal transition may be defined by the upper limit of the internal sphincter of the anus (between arrows in **a**), which is generally much thicker than the inner muscular layer of the *muscularis propria* of the rectum, or by the plane that intersects the superior border of the *puborectalis muscle* (between arrows in **b**), which may be asymmetric, as in the example depicted

The rectum ends distally at the anorectal transition. The anorectal transition may be defined by 2 anatomic landmarks: the first is an abrupt increase in thickness of the inner muscular layer, corresponding to the upper limit of the internal sphincter of the anus (Fig. 1a) [4]. The other is the superior border of the puborectalis—the sling or U-shaped portion of the levator ani muscle complex (or “pelvic diaphragm”)—which is anchored anteriorly to the inferior pubic ramus on each side of the symphysis pubis and posteriorly to the anococcygeal raphe (Fig. 1b) [5, 6].

**Fig. 2:**
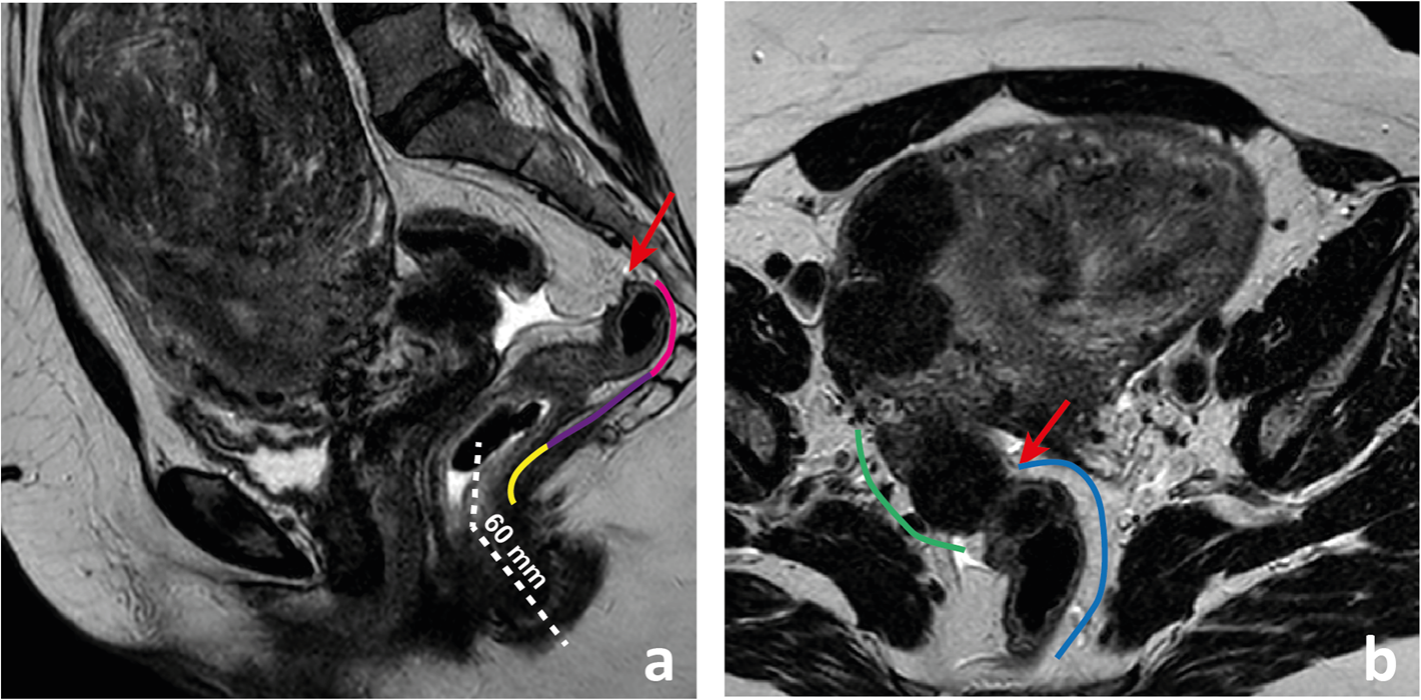
The upper limit of the rectum corresponds on MR imaging to the point of inflexion between the more vertical rectum and the more horizontal sigmoid “take off” (arrow in **a**). The sigmoid starts at another more horizontal inflection, better visualised in the (oblique) axial plane (arrow in **b**). The low rectum is the segment located < 6 cm above the anal verge and above the anorectal transition (lined in yellow in **a**); the mid-rectum is located between the low rectum and the plane of the lowest point of the anterior peritoneal reflection (lined in purple in **a**); the high rectum is the segment above it and below the sigmoid “take-off” (lined in pink in **a**). The sigmoid “take-off” or rectosigmoid transition is lined in blue in **b**, while the actual sigmoid is lined in green

The definition of the upper limit of the rectum is an intraoperative definition, corresponding to the lower limit of the large bowel that can be mobilised away from the spinal column. On MR imaging, it may correspond to the point of inflection between the more vertical rectum and the more horizontal rectosigmoid or “sigmoid take-off” [7–9] (Fig. 2a). The actual sigmoid starts at another more horizontal inflection, away from the lesser pelvis (Fig. 2b).

The rectum may be divided into 3 segments: upper, mid and low. The upper rectum is located above the lower limit of the anterior peritoneal reflection (see below) (Fig. 2a) [8]. The low rectum is surgically defined as the portion of the rectum that is less than 6 cm from the anal verge, visible on sagittal T2-WI as the lower limit of hypointense skin change (Fig. 2a) [10, 11]. The middle rectum is in between these two segments (Fig. 2a).

##### Keep in mind

For standardization purposes, we recommend rectal cancer location measurements to be made based on the central axis of both the anal canal and rectum (Fig. 2a). Rectum definitions should be clear to the whole multidisciplinary team given older definitions are still frequently utilised. The latter include the distal-most 16 cm, 15 cm or 12 cm of the large bowel and the segment spanning from the anorectal transition to S3 or to the promontory [12].

#### The rectal wall

**Fig. 3:**
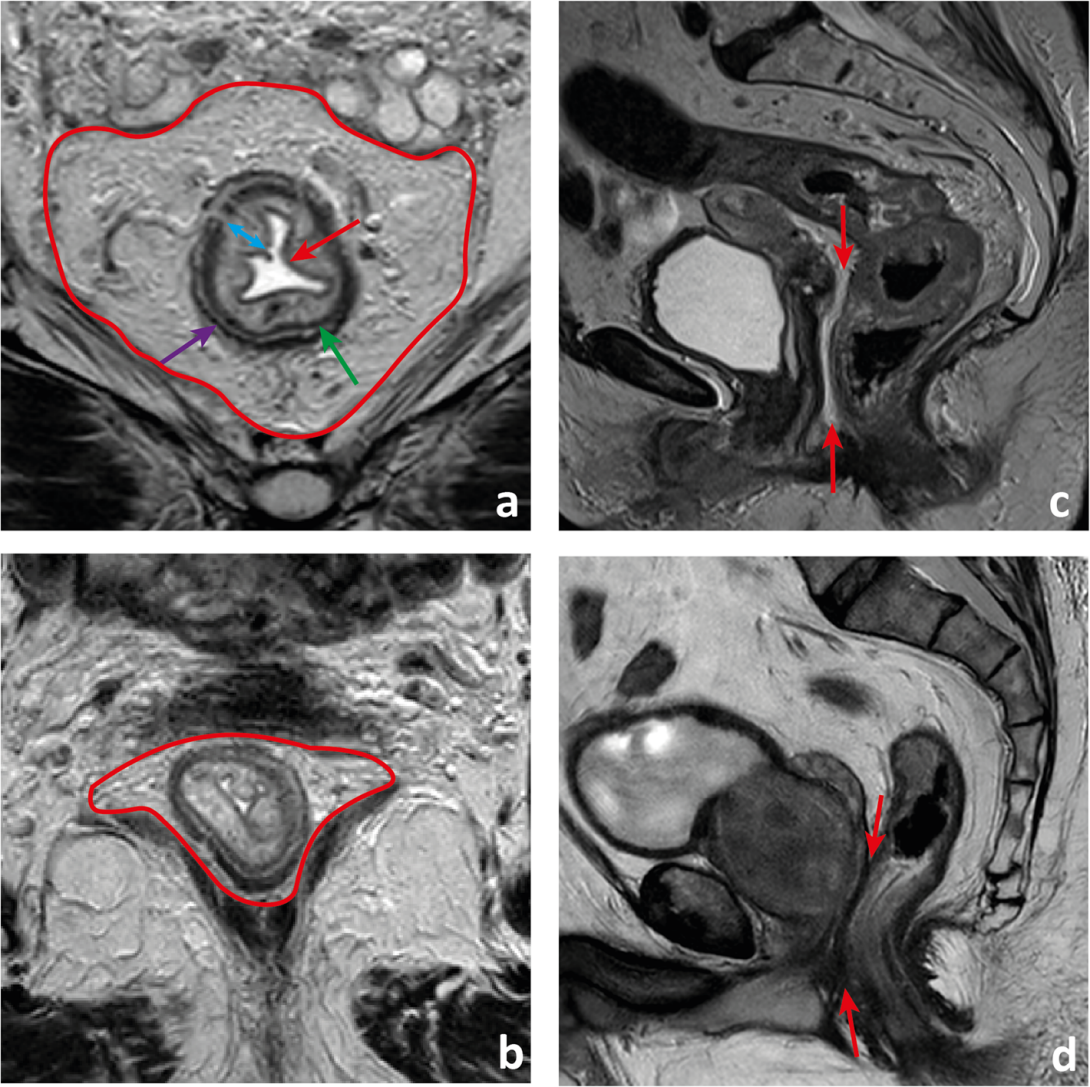
The rectal wall. The mucosa is visible on T2-weighted imaging as a regular thin (≈ 1 mm) hypointense line delimiting the lumen (red arrow in **a**). The submucosa is hyperintense and of variable thickness (double-headed blue arrow in **a**). It may not be visible when the rectum is distended. The *muscularis propria* is composed of an inner circular layer (green arrow in **a**) and an outer longitudinal layer (purple arrow in **a**) which may be separated by a thin layer of fat, as in the example shown. Lying externally to it is a cushion of mesorectal fat (delimited in red in **a**) that tappers inferiorly (delimited in red in **c**) and anteriorly, where it can be thin (between arrows in **c**) or even invisible (between arrows in **d**), in which case the mid/low rectum appears juxtaposed to the Dennonvillier fascia

Although peristalsis, pulsation, breathing and susceptibility from bowel air may cause image artefacts, in optimal conditions, the layers of the rectal wall should be clearly defined. The mucosa is a thin, dark regular line on T2 (Fig. 3a). It is underlined by the T2-hyperintense fat-rich submucosa which is highly variable in thickness from patient to patient and also according to the degree of distension of the rectum (Fig. 3a). The muscularis propria is dark on T2 (similarly to skeletal muscle) and is composed of an inner circular layer and an outer longitudinal layer (Fig. 3a). Once again, its thickness varies according to the degree of distension of the rectum. The longitudinal layer of the muscularis propria is covered by the fat-rich mesorectum, which is highly variable in thickness according to body composition and unevenly distributed (Fig. 3a–d). Anteriorly, it may be very thin or even not visible, particularly in the 2 cm above the anorectal transition (Fig. 3c, d) [12].

##### Keep in mind

**Fig. 4:**
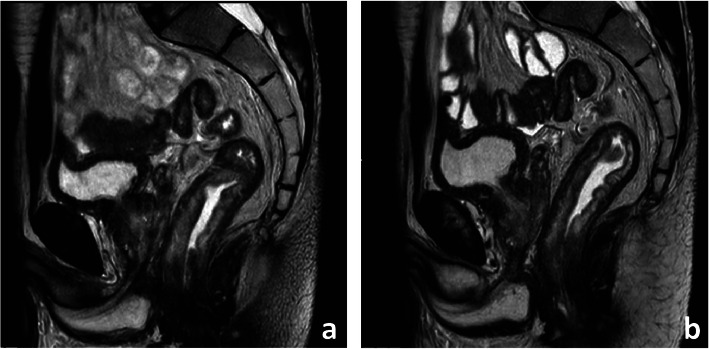
Sagittal T2-weighted images before (**a**) and 5 min after (**b**) IV administration of a spasmolytic agent (butylscopolamine 20 mg). Notice how the walls of the rectum and small bowel are much better defined due to limited peristalsis

A small enema before MR imaging may minimise susceptibility from bowel air [13]. Spasmolytic agents such as butylscopolamin or glucagon significantly reduce artefacts from peristalsis and may be administered to improve image quality in the absence of contraindications (Fig. 4) [13]. Our rectal cancer staging acquisition parameters at 1.5T are presented in Table 1.

**Table 1:** MR acquisition parameters at a 1.5 T Ingenia Philips Healthcare®, Best, The Netherlands equipment

Parameter	Oblique axial T2-weighted turbo spin-echo*	Oblique coronal T2-weighted turbo spin-echo	Sagittal T2-weighted turbo spin-echo	Single-shot spin-echo echo-planar diffusion-weighted^**+**^	2D T1-weighted gradient-echo
**Echo time (ms)**	85	100	100	90	10
**Repetition time (ms)**	5692	2000	2660	4288	683
**Echo train length**	18	16	21	–	–
**Slice thickness (mm)**	3	3	3	5	5
**Gap (mm)**	0.3	0.3	0	0	1
**Matrix**	416 × 465	200 × 179	252 × 223	76 × 65	292 × 300
**Field-of-view (mm)**	250 × 328	160 × 160	200 × 200	200 × 200	290 × 348
**In-plane resolution (mm**^**2**^**)**	0.6 × 0.7	0.8 × 0.8	0.8 × 0.9	2.6 × 3.01	1 × 1.16
**Signal averages**	1	2	2	7	1

*Oblique axial scans are acquired perpendicular to the long axis of the rectal wall at tumour location

^+^Spectral pre-saturation with inversion recovery is utilised for fat saturation. *B* values of 0 and 1400 s/mm^2^ are employed

#### The envelope

**Fig. 5:**
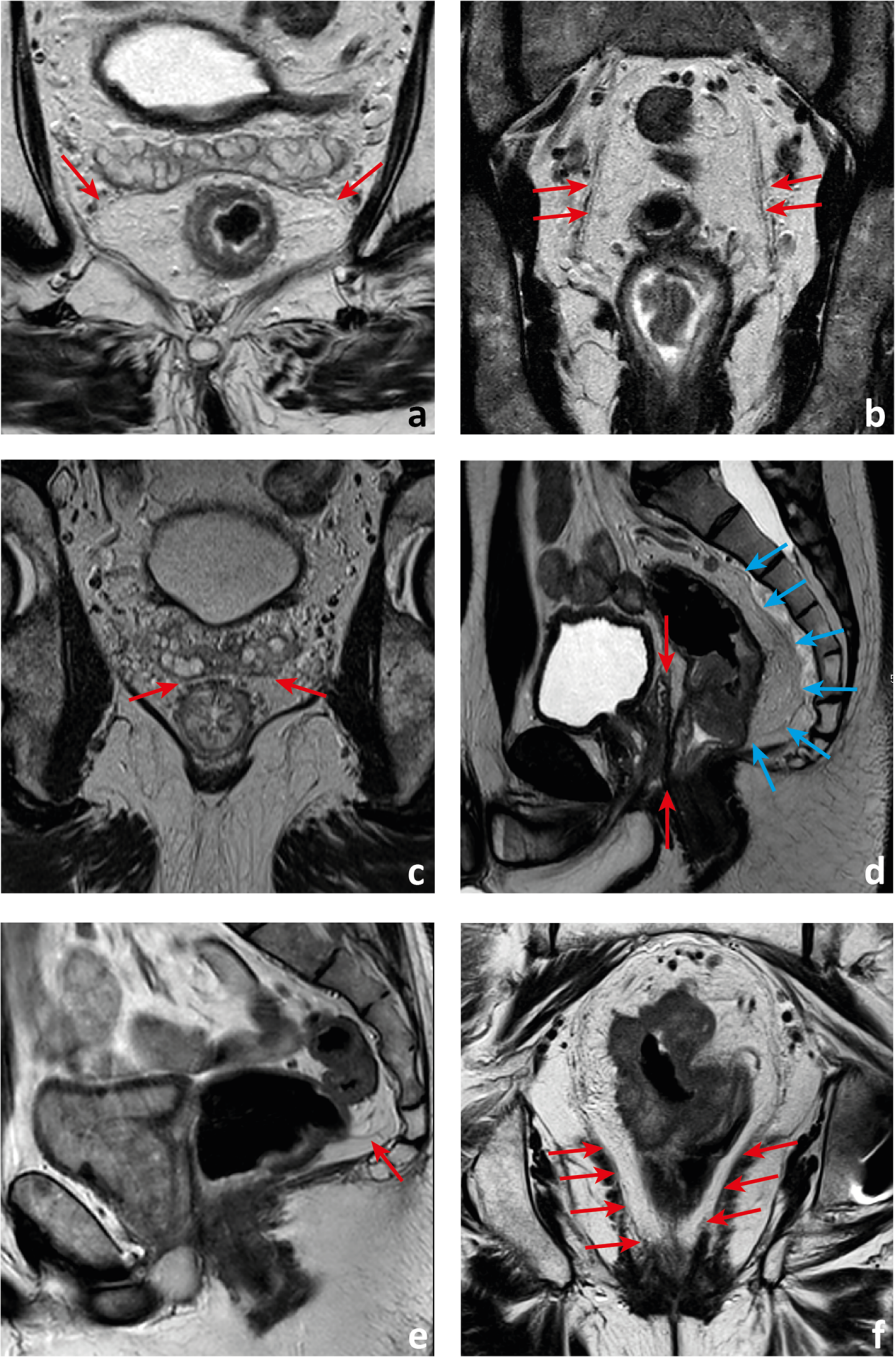
The mesorectal fascia may present with gaps, particularly anterolaterally (arrows in **a**). It is a multilayered envelope and as such multiple hypointense lines may be apparent (arrows in **b**). Anteriorly, in between the neurovascular bundles, extending from the peritoneal reflection down to the pelvic floor, the mesorectal fascia is juxtaposed to the Dennonvillier fascia (between red arrows in **c** and **d**). Posteriorly, it is juxtaposed to the presacral fascia (blue arrows in **d**). Sometimes, the presacral compartment is divided by the Waldeyers fascia (arrow in **e**) into superior and inferior compartments. Inferiorly, the mesorectal fascia tappers and is “lost” within the intersphincteric plane (arrows in **f**)

The rectum is out of the peritoneal cavity, and its serosa—the mesorectal fascia—is displaced radially to contain a large fatty cushion—the mesorectum. The mesorectal fascia is usually described as a pencil-drawn hypointense line on T2. However, it is a multi-layered envelope that presents with gaps, particularly at its lower antero-lateral aspect, which means that at some points it may not be visible and we must extrapolate its expected position (Fig. 5a) [14]. Also, it is juxtaposed to the parietal fascias, which contain the autonomic nerves [14]. As such, multiple pencil-drawn T2-hypointense lines may be apparent, in which case the innermost should be considered (Fig. 5b). The virtual space between the mesorectal fascia and the parietal fascias is the holy plane of rectal cancer surgery—the plane through which the surgeon must perform sharp dissection to obtain optimal oncologic results with minimal bleeding and autonomic nerve damage-related morbidity.

The parietal fascias have different names according to location. Anteriorly, in between the anterolateral neurovascular bundles and extending craniocaudally from the pelvic floor to the peritoneal reflection is the trapezoidal-shaped Dennonvillier fascia (Fig. 5c, d) [15]; posteriorly, covering the sacrum, is the presacral fascia (Fig. 5d); and extending between the mesorectal fascia and the presacral fascia at the level of S2/S4 is the Waldeyer’s/rectosacral fascia (Fig. 5e). It divides the retrorectal space into superior and inferior compartments [14, 16–18].

**Fig. 6:**
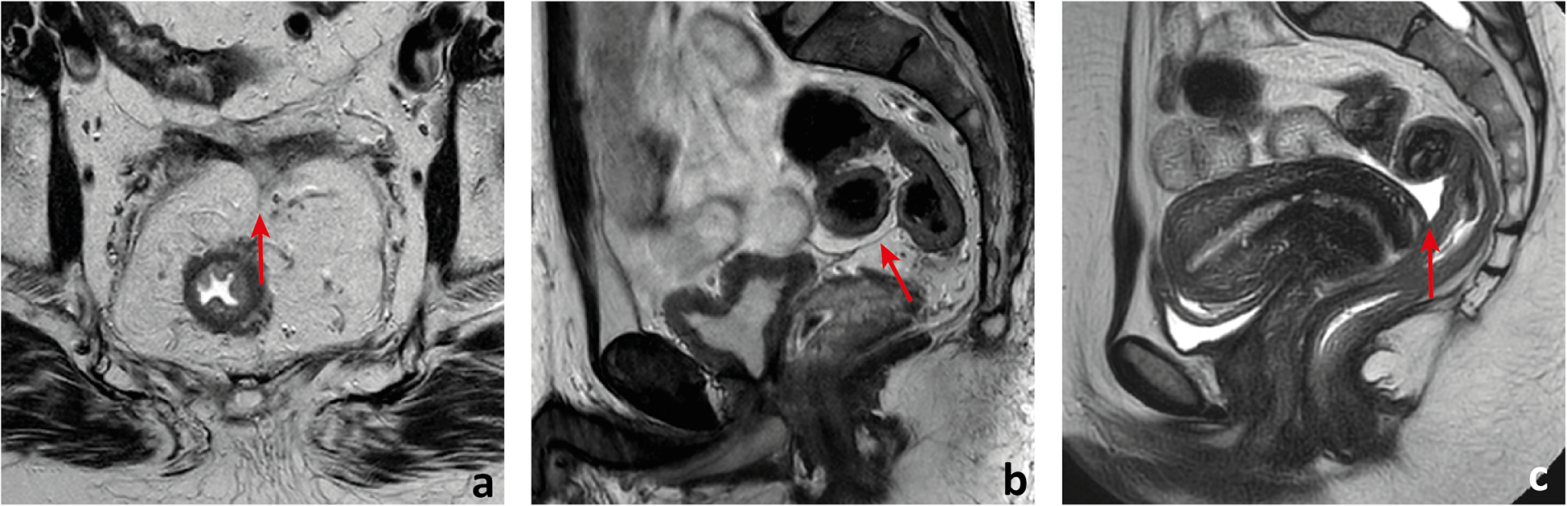
The peritoneal reflection, when visible, may present with a seagull or V-shaped appearance on the (oblique) axial plane (arrow in **a**). The sagittal plane is depicted as a hypointense line extending from the rectal wall to the roof of the mesorectal fascia (arrow in **b**). It is more easily identified whenever there is a small amount of peritoneal effusion, which will accumulate immediately above it (arrow in **c**)

Caudally, the mesorectal fascia is in continuity with the intersphicteric plane (Fig. 5f) [18]. Cranially, it is in continuity with the peritoneal reflection, which is lower anteriorly, of intermediate height laterally and higher posteriorly. The most relevant reference while staging rectal cancer is the anterior peritoneal reflection, which we should look for in the plane immediately below any pelvic small bowel loops and the sigmoid take-off. Its appearance on (oblique) axial plane is that of a seagull or V-shaped T2-hypointense line infolding (Fig. 6a). On mid-sagittal plane, this T2-hypointense line extends roughly horizontally from the anterior rectal wall to the roof of the mesorectal fascia, most commonly at the level of the torus uterinus in women or superior bladder in men (Fig. 6b) [19, 20]. The posterior peritoneal reflection is located most commonly 1 to 4 cm below S1–S2 and corresponds to the upper limit of the rectum [21, 22]. It is not easily identified.

##### Keep in mind

The anterior peritoneal reflection can easily be identified whenever there is a small amount of free peritoneal fluid because in the supine position it will accumulate at the deepest point of the rectovesical or rectouterine pouch, exactly where the peritoneum “reflects” (Fig. 6c). The height of the anterior peritoneal reflection is variable, and it may not be visible in > 10–25% of cases [19, 20].

#### The blood supply

**Fig. 7:**
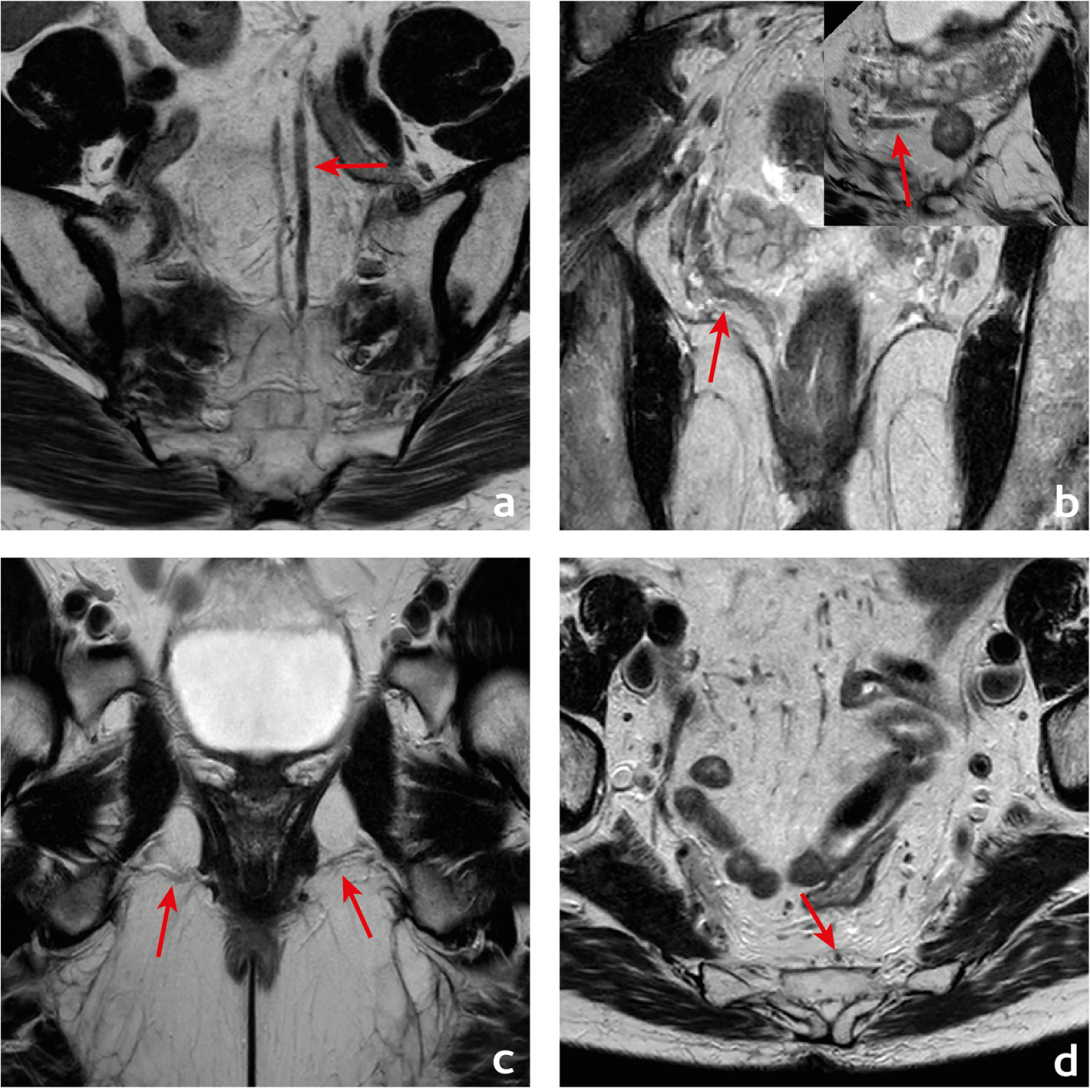
The rectum’s arterial supply has 3 to 4 routes. The main route is the superior rectal artery (arrow in **a**). The middle rectal arteries are inconsistent but may be present in up to 57% of cases. In **b**, a large calibre right middle rectal artery (arrow) arising from the internal iliac artery pierces the mesorectal fascia at its anterolateral aspect. The inferior rectal arteries originate from the internal pudendal arteries, are usually small in caliber and cross the ischioanal fossae towards the anal canal (arrows in **c**). The middle sacral artery courses down along the lumbosacral vertebrae, within the presacral space (arrow in **d**)

**Fig. 8:**
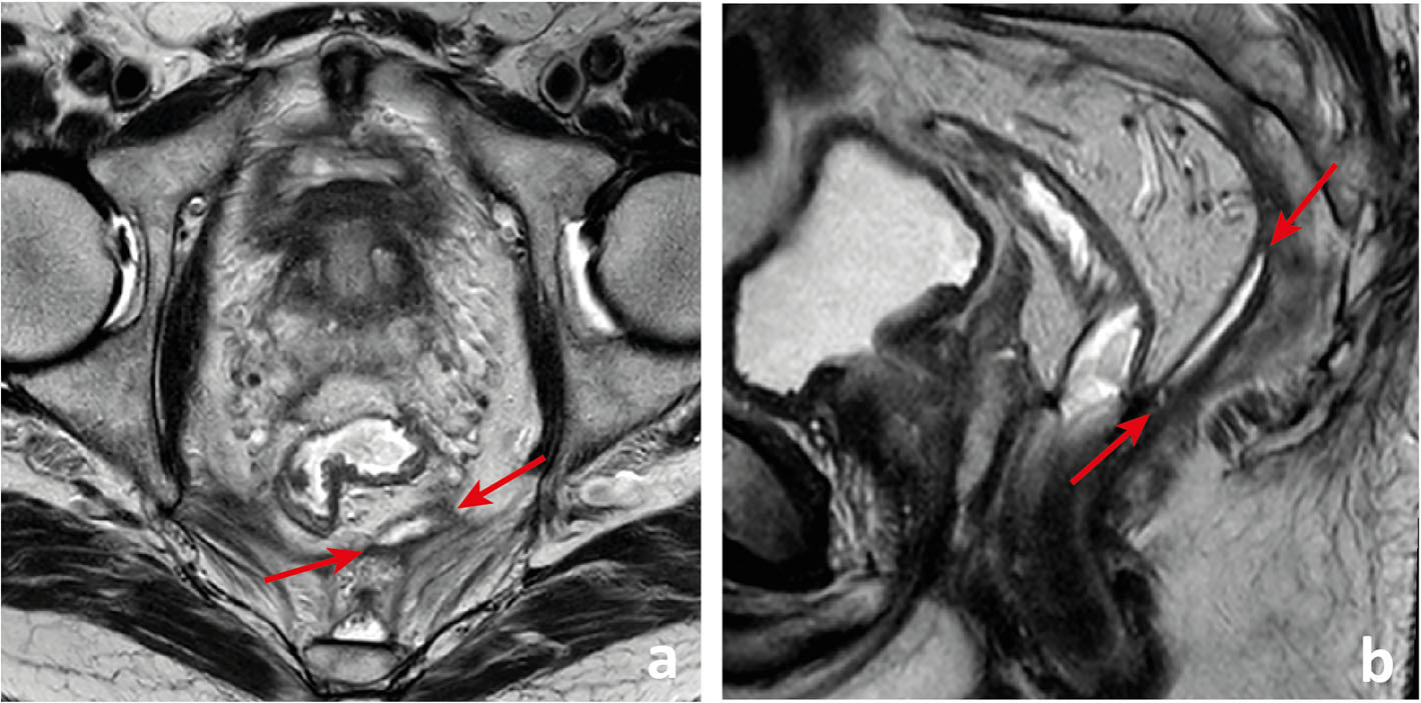
Patient who underwent low anterior resection for rectal cancer presented with a posterior colorectal anastomotic leak which became a chronic fluid collection (between arrows in **a** and **b**)

Arterial blood arrives at the rectum through 4 routes. The main route is the superior rectal artery, which is the continuation of the inferior mesenteric artery when it crosses the left common/internal iliac artery (Fig. 7a) [22]. The middle rectal arteries are inconsistent (present in 36–57% of cases) and highly variable. They may be unilateral, double or treble and may arise from the internal iliac or one of its branches [22–24]. They usually reach the mesorectum through either its antero-lateral or postero-lateral aspect, roughly 6 cm above the pelvic floor (Fig. 7b) [22]. The inferior rectal arteries originate from the internal pudendal arteries below the levator ani muscle, cross the ischioanal fossa anteromedially and irrigate the distal rectum, anal canal and internal and external anal sphincters (Fig. 7c) [24]. The middle sacral artery courses inferiorly along the surface of the lumbo-sacral vertebrae, within the presacral space and usually contributes with small vessels to the posterior surface of the rectum (Fig. 7d) [24]. The 4 rectal arterial routes communicate extensively through intramural anastomosis. However, in the posterior and inferior portion of the low rectum, there is a relatively vessel-deficient area which may explain why most anastomotic leaks after low anterior resections (LAR) occur in this location (Fig. 8) [22].

The venous drainage takes place through both an outer muscularis plexus that extends longitudinally from the anterior peritoneal reflexion down to the *levatior ani*, and an inner submucosal plexus which runs through the whole extension of the rectum and continues down to the anus [22]. Both plexus anastomose superiorly give rise to the superior rectal vein, draining the roughly upper 2/3 of the rectum into the portal circulation through the inferior mesenteric vein [22]. Inferiorly, the inner plexus becomes the inferior rectal vein. A middle rectal vein may be found in about 32.6%, usually unilaterally and rarely accompanied by a middle rectal artery. Inferior and middle rectal veins drain roughly the distal 1/3 of the rectum into the systemic circulation through the internal iliac vein [22, 25].

##### Keep in mind

Surgeons may find information on the presence of middle rectal artery(ies) and vein(s), their route and calibre useful to prevent unexpected bleeding, and as such, we should provide that information on staging reports. The fact that the lower 1/3 of the rectum drains into the systemic circulation rather than the portal circulation may at least partially justify the higher rate of lung metastasis in patients with low rectal cancer [26].

#### The lymphatic drainage

**Fig. 9:**
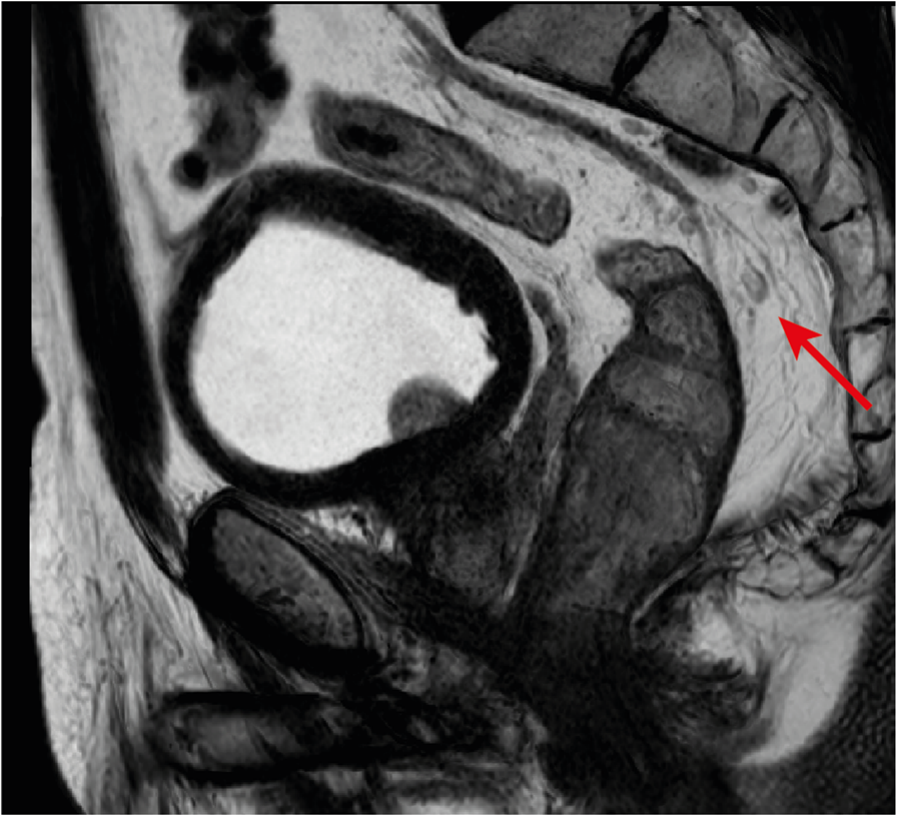
Normal distribution of lymph nodes in the mesorectum (sagital minimum intensity projection). Lymph nodes are more numerous and larger in the high posterior mesorectum (arrow points to the largest lymph node but a few more are visible upstream)

**Fig. 10:**
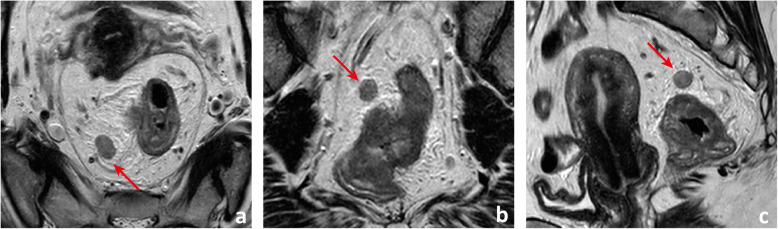
Patient staged as mrT3a/b mrN2b due to multiple bulky lymph nodes in the mesorectum of which one is depicted, measuring 13 mm in the long axis and 10 mm in the short axis. The patient underwent surgery and was a pT3N0. While carefully reviewing the lymph node appearance on T2-WI, we notice they all presented with well-defined borders, homogeneous signal intensity and a preserved chemical shift effect

The intramural lymphatic network drains to extramural lymphatics that in general follow the course of blood vessels [24]. Along the course of lymphatics within the mesorectum, we find a variable number of lymph nodes (mean 6.8 to 73.7 in surgical TME specimens with varied disease processes, surgical technique and pathology processing) [27–30]. Their size ranges between 2 and 10 mm in normal subjects, and they are more numerous and larger in the upper and posterior mesorectum (Fig. 9) [27–30]. Normal/reactive lymph nodes on T2-WI MR imaging appear homogeneous in 48 to 88% and with smooth, sharply demarcated borders in 80 to 94% of cases [31, 32]. In approximately 70% of cases, they will show a smooth and regular, uninterrupted chemical shift effect on T2-WI in the phase encoding direction, likely the result from the sharp interface between the water-rich subcapsular sinus and the mesorectal fat (Fig. 10) [32]. Lymph nodes located within the mesorectum, along the inferior and superior rectal vessels, along the inferior mesenteric vessels and along the internal iliac vessels and their branches are considered regional.

##### Keep in mind

Although considered regional by the AJCC, internal iliac lymph nodes, commonly referred to as “lateral pelvic lymph nodes”, are outside of the “circumferential margin of resection” in rectal cancer surgery and as such pose different management challenges (see below).

### Staging rectal cancer

#### The primary—T stage

**Fig. 11:**
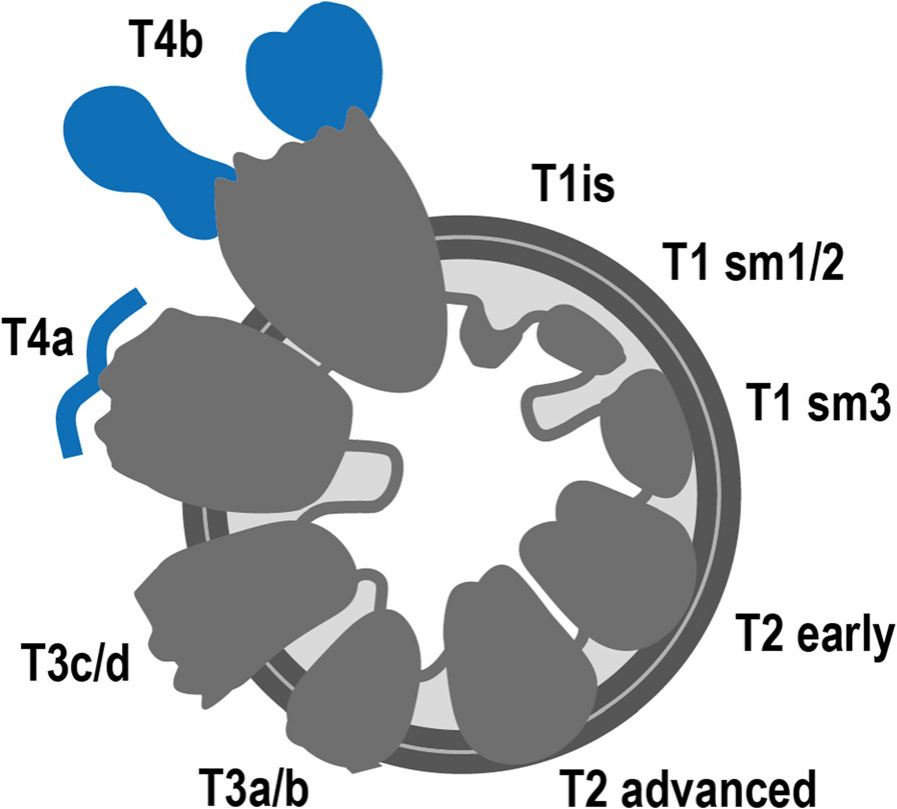
T stages and substages. T1is: tumour in situ; T1sm1/2: tumour invades the submucosa, but not its full thickness; T1sm3: tumour invades the full thickness of the submucosa; T2 early: tumour focally invades the *muscularis propria*; T2 advanced: tumour invades the full thickness of the *muscularis propria*; T3a: tumour extends < 1 mm into the mesorectal fat; T3b: tumour extends between 1 and 5 mm into the mesorectal fat; T3c: tumour extends between 5 and 15 mm into the mesorectal fat; T3d: tumour extends > 15 mm into the mesorectal fat; T4a: tumour invades the peritoneum; T4b: tumour invades adjacent organs

**Fig. 12:**
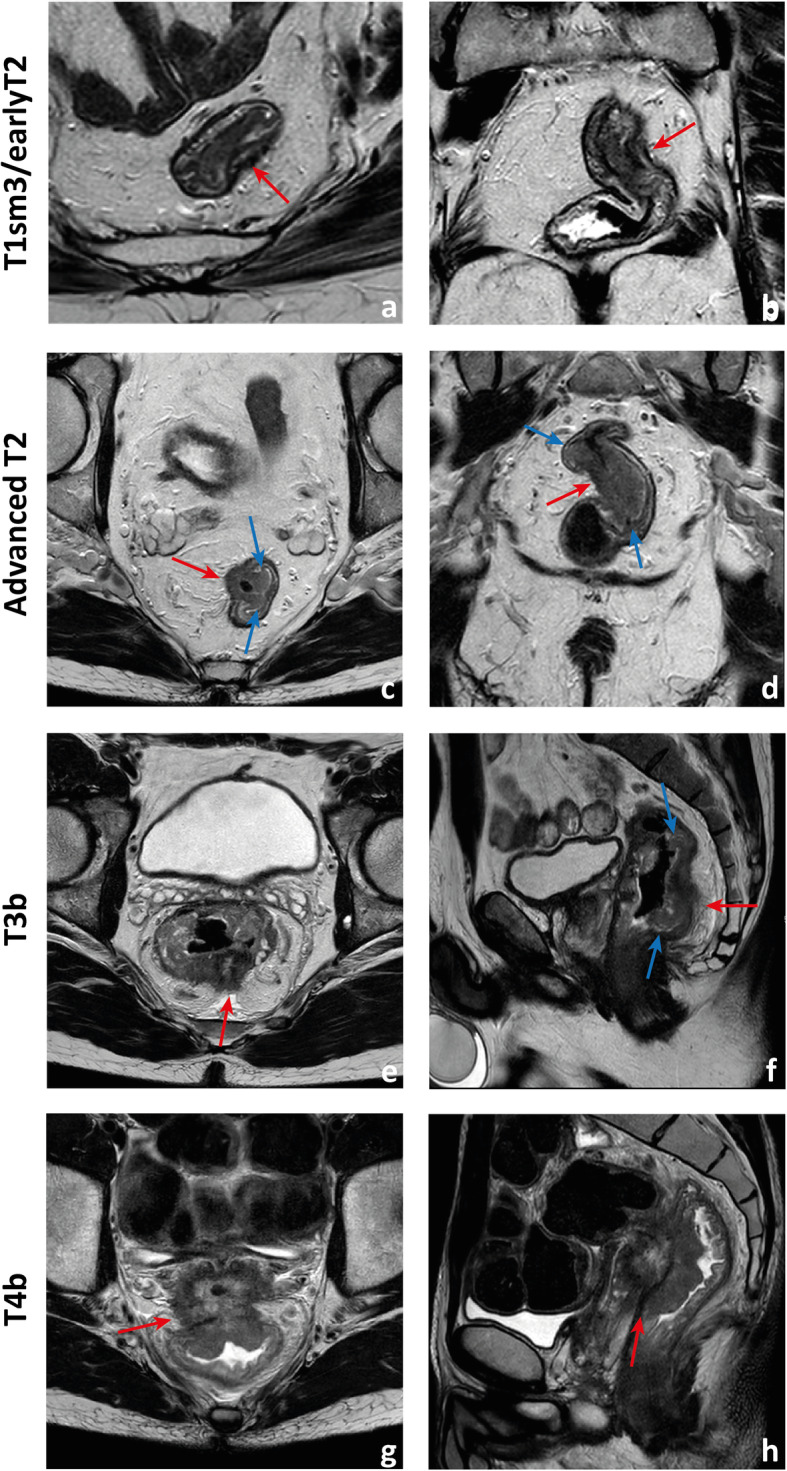
Examples of differently T-staged tumours. **a**, **b** Arrows point to an early polypoid tumour occupying the full depth of the submucosa centrally and therefore staged clinically as mrT1sm3/early T2. Total mesorectal excision was performed, and at pathology, it corresponded to a T1sm3. **c**, **d** This ulcerated tumour extends in depth through the full thickness of the *muscularis propria* and was therefore staged as an MR advanced T2 (red arrows), which was confirmed at pathology. Notice how the periphery of the tumour overhangs the rectal wall both in the axial and coronal planes (blue arrows). **e**, **f** This mid-high, sub-circumferential rectal cancer extends 4 mm into the mesorectal fat at its most central area (red arrows) and was therefore staged as T3b. Notice the overhanging edges on the sagittal plane (blue arrows in **f**). **g**, **h** An anterior mid-high rectal cancer crosses the Denonvillier fascia and the peritoneal reflection to invade the seminal vesicles in this male patient, corresponding to an mrT4b. The patient underwent chemoradiation and is currently under palliative treatment due to distant metastatic disease

We find reading the endoscopy report carefully to aid in the interpretation of the images—we should check whether the gastroenterologist found a suspicious focus in a benign polyp or a clearly malignant infiltration of the rectal wall and pay special attention to the lesions’ shape description, estimated location, size and circumference of involvement. Also, we believe DWI may help locate small primary tumours, given on high *b* value images they will present most commonly with high signal intensity, but high-resolution T2-weighted imaging (T2-WI) perpendicular to the long axis of the tumour is the pillar of T staging given it provides the highest rectal wall layer detail. The maximum depth of tumour invasion will determine the MR imaging T stage (mrT) (Figs. 11 and 12).

Although NCCN guidelines consider any tumour ≥ mrT3 eligible for neoadjuvant therapy, in many European countries, mrT3 sub-classification according to the depth of invasion beyond muscularis propria is incorporated in the decision-making framework (Fig. 11) [33, 34]. Neoadjuvant therapy is, according to European (ESMO) guidelines, reserved for tumours > mrT3b which pose a higher risk of local recurrence (23.6% vs 10.4%) [34]. Reported MR imaging accuracy for the assessment of T stage is variable, ranging from 66 to 94% using pathology as a reference standard [35, 36]. The main difficulty is the differentiation between T2 and T3, and discriminating between the two is particularly relevant on low tumours given the much thinner cushion of mesorectal fat and consequent proximity to sphincter complex and middle pelvic compartment structures [35, 36]. T2 tumours may present with desmoplastic reaction leading to overstaging as T3. Desmoplasia tends to present as thin spiculae of T2-hypointensity surrounding invaded muscularis propria, whereas T3 tumours usually present with a broad-based or nodular T2-intermediate-signal front at mesorectal fat [37].

##### Keep in mind

The maximum depth of invasion tends to occur at the tumour’s centre, and in large tumours, orientation of the slices perperdicular to it may be the most useful. On the other hand, it is common for the tumour’s periphery to overhang the rectal wall into the lumen (Fig. 12c, d, f).

#### The circumferential margin of resection

**Fig. 13:**
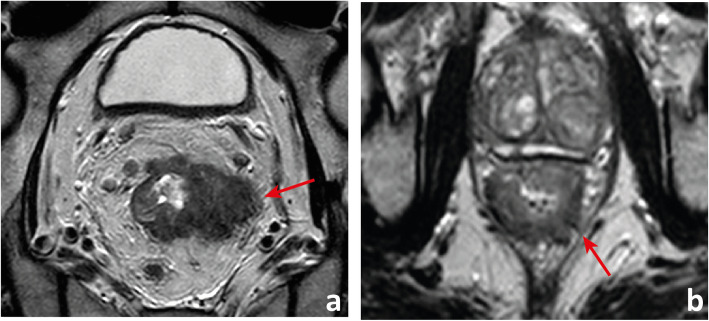
Circumferential margin of resection involvement. **a** A T3d tumour invades the mesorectal fascia on the left side (arrow). **b** A 1–2 mm fat plane between this mrT3b low rectal cancer and the sphincter complex (arrow) is observed and as such the circumferential margin of resection may be considered free

The circumferential margin of resection (CRM) is the plane where surgeons must dissect in order to perform a standardised total mesorectal excision (TME). It corresponds to the full circumference of the mesorectal fascia and, in tumours extending down into the anal canal, to the intersphincteric plane, with which the mesorectal fascia is in continuity inferiorly. The minimum distance between the tumour and the circumferential margin of resection on high-resolution T2-WI acquired perpendicular to its long axis should be recorded, as well as its location and extent. CRM is considered involved if the tumour lies < 1 mm from it (Fig. 13a) [38]. Although MR imaging was considered very accurate and reliable at predicting a clear margin in 2 seminal studies by Brown et al. and Beets-Tan et al., in a large meta-analysis, the sensitivity and specificity for margin involvement using a 1-mm cutoff were of only 76% and 88% respectively [39–41]. Margin involvement at MR imaging is associated with higher local recurrence rates (20% vs 7.1%), worse overall survival (42.2% vs 62.2%) and disease-free survival (47.3% vs 67.2%) [39, 42]. It is considered an indication for neoadjuvant therapy by both NCCN and ESMO guidelines [33, 34].

#### The extramural venous invasion

**Fig. 14:**
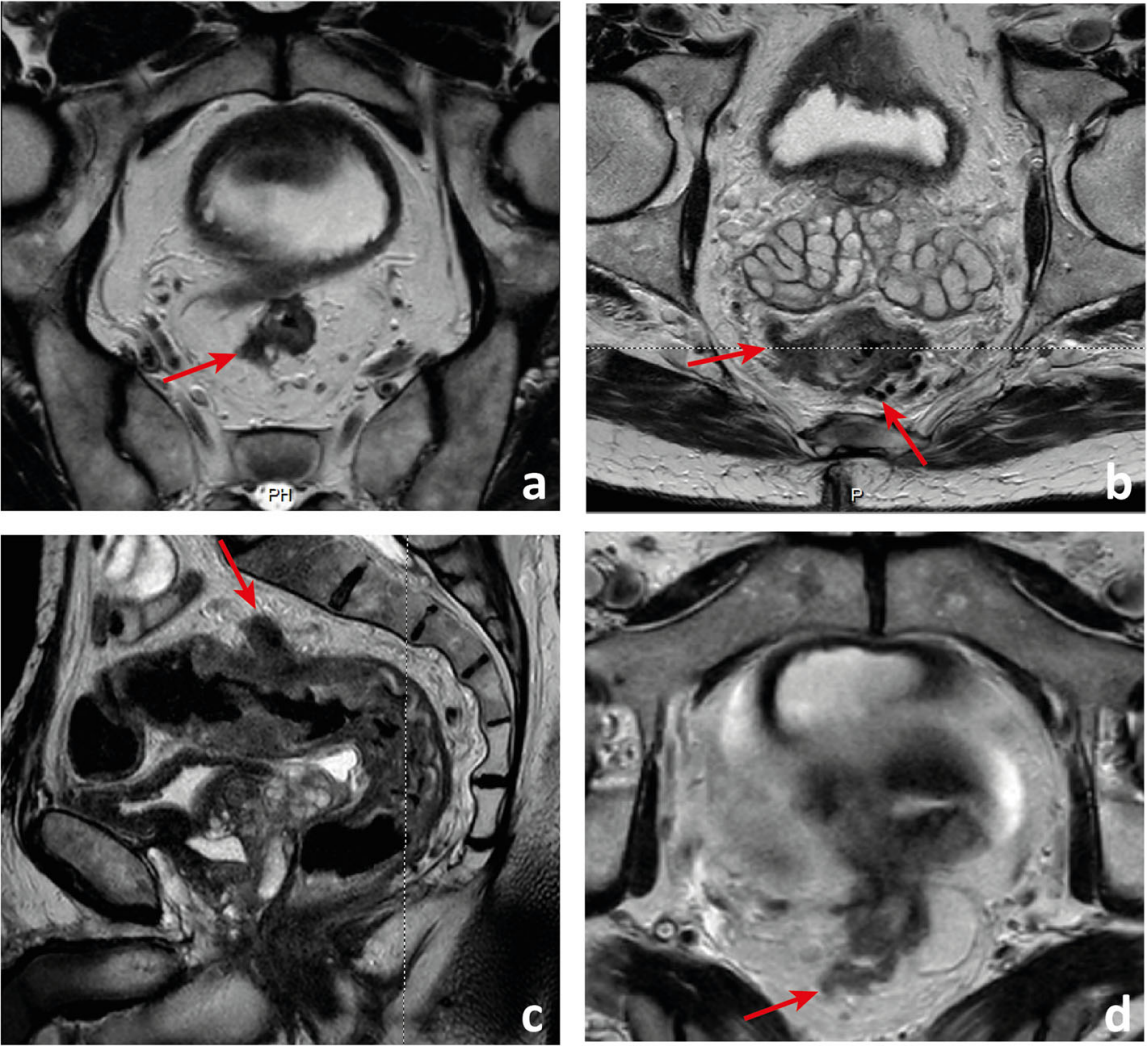
MR extramural venous invasion (mrEMVI) is characterised by the presence of intermediate signal intensity tumour signal within vessels, which may be expanded (arrows in **a** to **d**) with (arrow in **a**) or without contour irregularity

Extramural venous invasion (EMVI) on the histopathology of rectal cancer surgery specimens is an independent predictor of local recurrence, distant recurrence and worse overall survival [43]. MR imaging may detect EMVI with low sensitivity (62%) but high specificity (88%), using pathology as the gold standard [43]. It is depicted as intermediate signal intensity within vessels, with or without associated expansion and contour irregularity. It is generally in continuity with the primary tumour, but discontinuous mrEMVI may also be observed. The likelihood of mrEMVI may be graded based on an ordinal scale by Smith et al., grades 3 and 4, characterised by the presence of intermediate signal intensity within vessels (Fig. 14), being associated with a lower relapse-free survival (35% vs 74%) [43, 44] and a higher risk of synchronous (OR 5.68; 95 CI 3.75–8.61) and metachronous (OR 3.91; 95 CI 2.61–5.86) metastasis [45]. Its presence is considered an indication for neoadjuvant therapy according to ESMO guidelines [34]. It is not part of the NCCN guidelines [33].

#### Lymph node involvement and tumour deposits

**Fig. 15:**
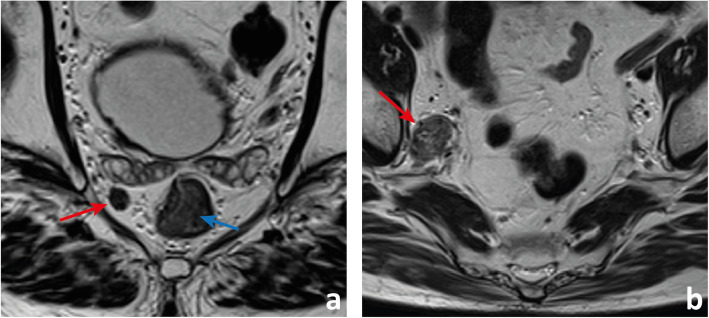
**a** This patient presents with a polypoid tumour (blue arrow in **a**) located 7 cm above the anal verge. Tumour is underlined by a clear, uninterrupted, hyperintense submucosal fat plane and may as such be staged as mrT1 sm1/2. The patient also presented with a hypointense round lymph node in the mesorectum (red arrow), at tumour plane, 10 o’clock, with a slightly irregular contour anterolaterally. A very bulky, irregularly contoured, heterogeneous lymphadenopathy was found in the obturator space of the right lateral pelvic sidewall. The patient was selected for chemoradiation therapy

In rectal cancer patients, nearly 48% of positive lymph nodes are ≤ 5 mm. As such, size criteria to identify nodal involvement are not reliable [27–30, 46]. Although normal lymph nodes are more numerous and larger in the upper and posterior mesorectum (Fig. 9), the incidence of nodal metastases is the same in anterior, lateral and posterior locations [27–30]. Regional positive lymph nodes in a more cranial location are associated with a higher risk of distant metastases [46]. Advanced T stage is associated with a higher number of positive lymph nodes, particularly small in size [46]. Most involved lymph nodes occur within the 1 cm proximal and the 1 cm distal to the tumour [47], and further spread occurs cranially in more than 90% of cases [48–50]. In the case of rectal cancers at the level of or below the peritoneal reflection, particularly ≥ T3, further spread may also occur to the lateral pelvic compartments [30, 51]. In fact, the lower the tumour, the more likely lateral spread is, going from 11.4% between 4 and 6 cm from the anal verge to 33.3% below 4 cm (Fig. 15) [51].

For nodes in the lateral pelvis, mostly size criteria have been tested, with variable cutoffs and variable outcomes [52–56]. According to research by the Lateral Node Study Consortium, in the particular case of cT3/4 low tumours, lateral lymph nodes with a short axis of at least 7 mm on staging MR have a significantly higher risk of lateral recurrence and lateral lymph node dissection in such cases may reduce lateral recurrences significantly [57]. For nodes > 3 mm in the mesorectum, contour irregularity, signal heterogeneity and interruption or absence of chemical shift artefact on T2-WI are the most reliable criteria for tumour infiltration [31, 32, 39]. No reliable criteria exist for nodes smaller than that [31, 39]. N staging based on MR imaging may be reported symmetrically to the TNM staging system.

**Fig. 16:**
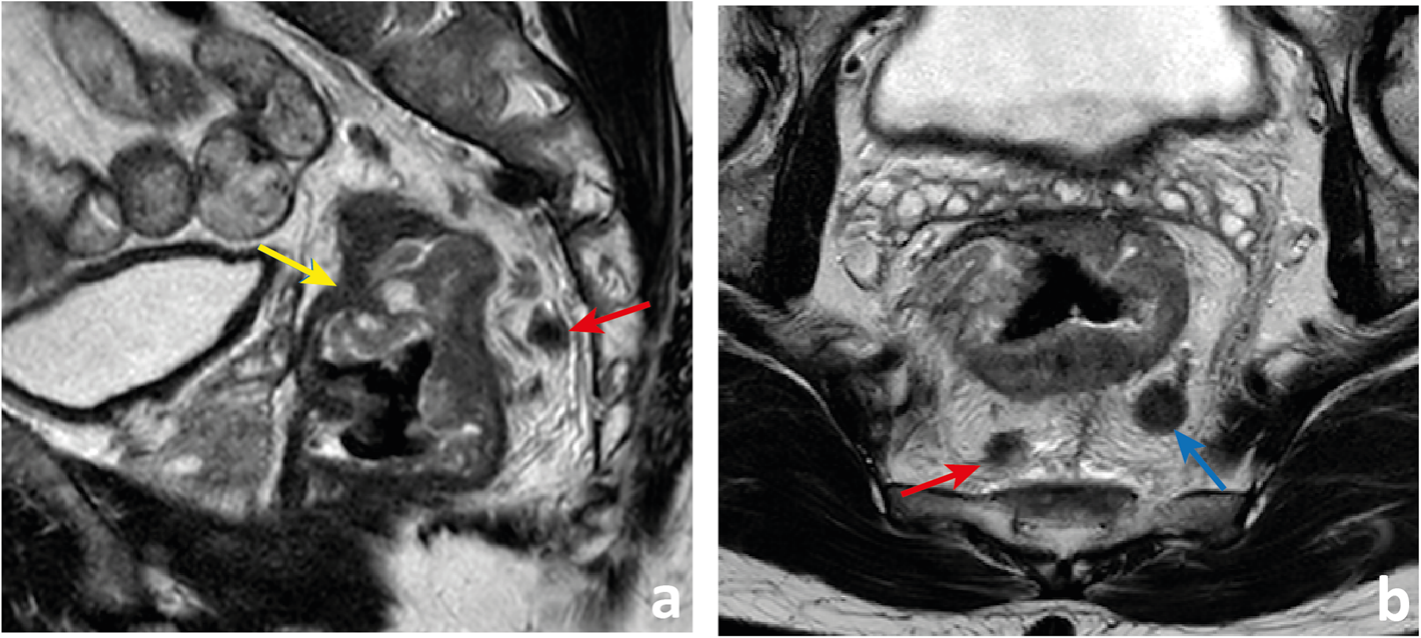
This patient with a large T3 MRF+ rectal cancer (yellow arrow in **a**) presents with a hypointense, highly irregular, intermediate-to-low signal intensity nodule in the mesorectum (red arrows in **a** and **b**) that does not interrupt the course of a vein but is irregular enough for us to admit the possibility of an extranodal deposit (mrENTD). The patient also presented with a round, hypointense slightly heterogeneous and slightly irregularly contoured lesion which we believe to be a lymphadenopathy (blue arrow in **b**)

Tumour deposits are defined according to AJCC 2018 as discrete tumour nodules in the rectal cancer pathology specimen within the lymphatic drainage area of the primary tumour without identifiable lymphatic, vascular or neural tissue, irrespective of their morphology [58]. If a vessel wall/remnant is identified, the lesion should be classified as lymphovascular invasion, further subclassified as either lymphatic or venous. If neural structures are identified, the lesion should be classified as perineural invasion. Tumour deposits are found in 3.3% of rectal cancer specimens without lymph node involvement, in which case N1c should be recorded (TNM classification), but when lymph nodes are positive, tumour deposits are not to be added to the total positive lymph node count. Lord et al. are currently studying the differentiation of extranodal tumour deposits (mrENTDs) from involved lymph nodes on MR imaging—the subject of an ongoing clinical trial named COMET [59]. Their imaging definition is quite different from the pathologic definition described above [59]. mr-ENTDs are described as comet-shaped nodules of tumour which appear to directly interrupt the course of a vein (Fig. 16) [59].

Although T stage now prevails, lymph node positivity on MR imaging is still considered an indication for neoadjuvant therapy according to NCCN guidelines, whereas ESMO guidelines admit surgery upfront for N positive patients without involvement of the circumferential margin of resection or mrEMVI, preferably ≤ T3a-b [33, 34].

##### Keep in mind

**Fig. 17:**
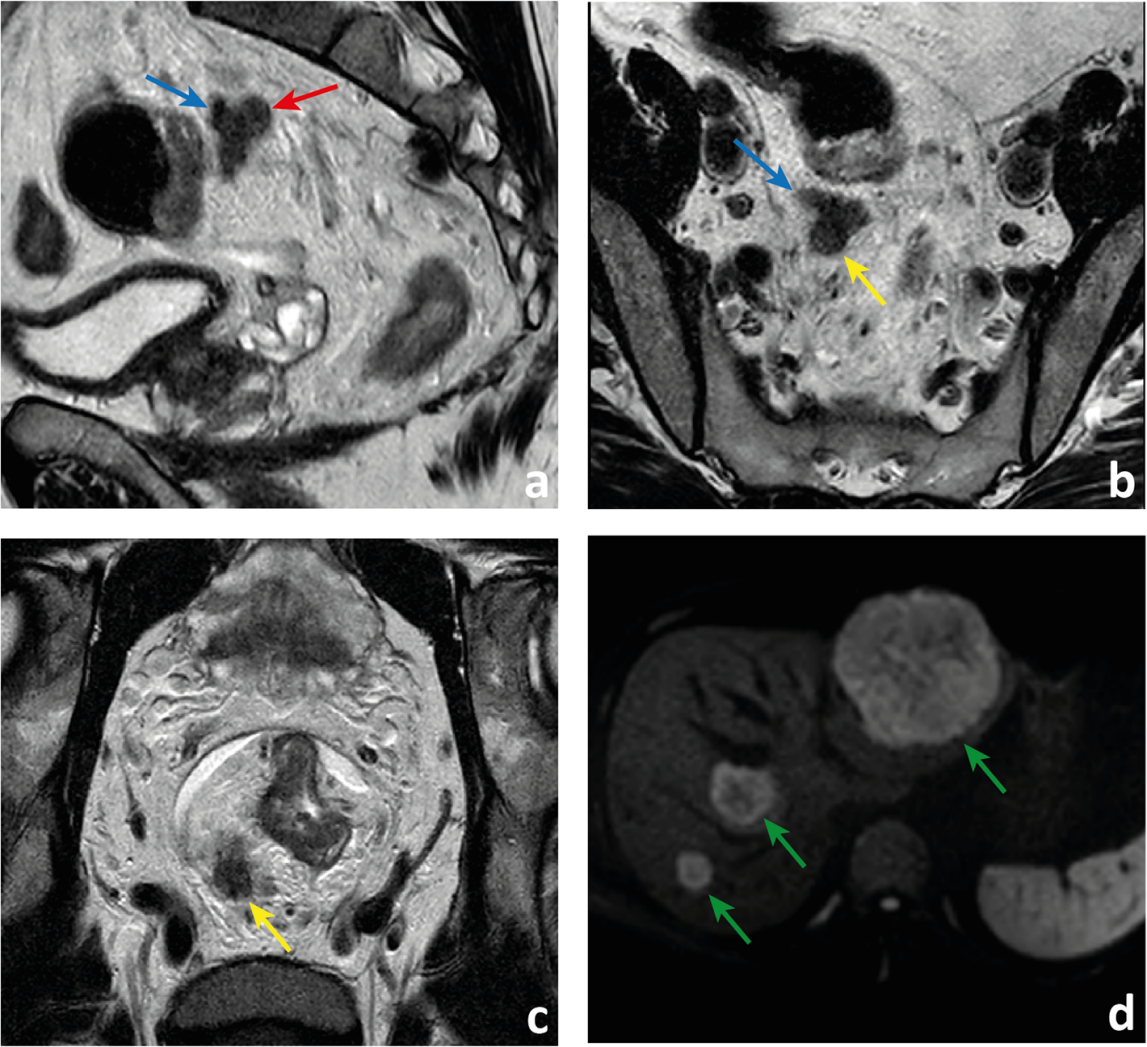
A patient with an mrT3d MRF+ high rectal cancer also presents with a very irregular hypointense lesion in the high mesorectum (red arrow in **a**) that appears to both interrupt the course of a vein suggesting mrENTD (blue arrow in **a**) and to grow within it suggesting discontinuous mrEMVI (blue arrow in **b**). It is also in continuity with a round, irregular and heterogeneous structure appearing to be a positive lymph node with extracapsular extension (yellow arrows in **b** and **c**). The patient presented with synchronous metastatic liver disease, visible on b900 DWI (green arrows in **d**)

In the authors’ perspective, the differentiation between positive lymph nodes with extracapsular extension, ENTDs and discontinuous EMVI on MR imaging may be difficult. Evidence suggests all of these entities entail a worse outcome compared to confined lymph node involvement. As such, pointing extranodal tumour on staging reports may be more important than matching its exact pathologic diagnosis (Fig. 17) [60].

### The specific scenarios

#### The very early rectal cancers

**Fig. 18:**
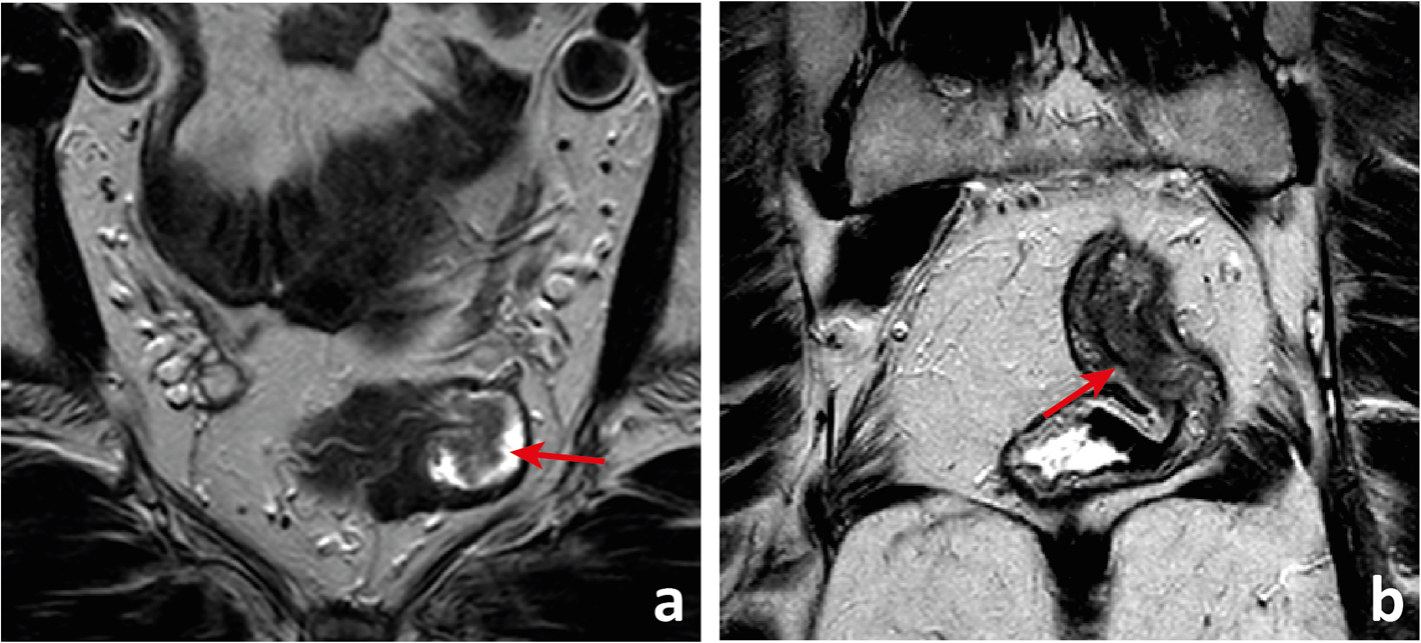
Two different patients with two different early rectal cancers are depicted in **a** and **b**. In **a**, a thin submucosal fat plane is observed between the intermediate signal intensity tumour and the tented *muscularis propria*. Tumour may therefore be staged as mrT1sm1/2. Pathology of the total mesorectal excision specimen revealed a pT1sm1; in **b**, the tumour abuts the inner circular layer of the *muscularis propria* and may therefore be staged as mrT1sm3/earlyT2. Pathology of the total mesorectal excision specimen revealed a pT1sm3

Very early rectal cancers, defined as low grade (G1/G2) cT1 cN0 cEMVI- tumours [33], can be subclassified based on the depth of invasion of the submucosa, tumours with < 1 mm of submucosal invasion having a practically null risk of lymph node metastasis [61]. This means transanal endoscopic microsurgery (TEM) can provide similar outcomes to TME in these patients, with much reduced morbidity and mortality [34]. The risk of lymph node metastases in cT1 tumours extending beyond 1 mm is relatively low (≅ 10%), particularly if G1/G2, V0, L0 [34, 62] and TEM may still be a reasonable upfront approach. MR imaging may have a role in the selection of these patients: Balyasnikova et al. could differentiate patients with either no evidence of submucosal invasion or a visibly spared ≥ 1 mm submucosa (mrT1 sm1/2)—considered eligible for TEM, from patients with no visibly spared submucosa or partially preserved muscularis (mrT1 sm3/earlyT2), with an accuracy of 89% and a good interobserver agreement (*k* = 0.74) [62]. Results of a larger UK multi-institutional trial (SPECC) are now awaited. MR imaging may also help assess lymph node involvement upfront and monitor recurrence after TEM. Fig. 18 depicts examples of both mrT1sm1/2 and mrT1sm3/earlyT2.

#### The low rectal cancers

**Fig. 19:**
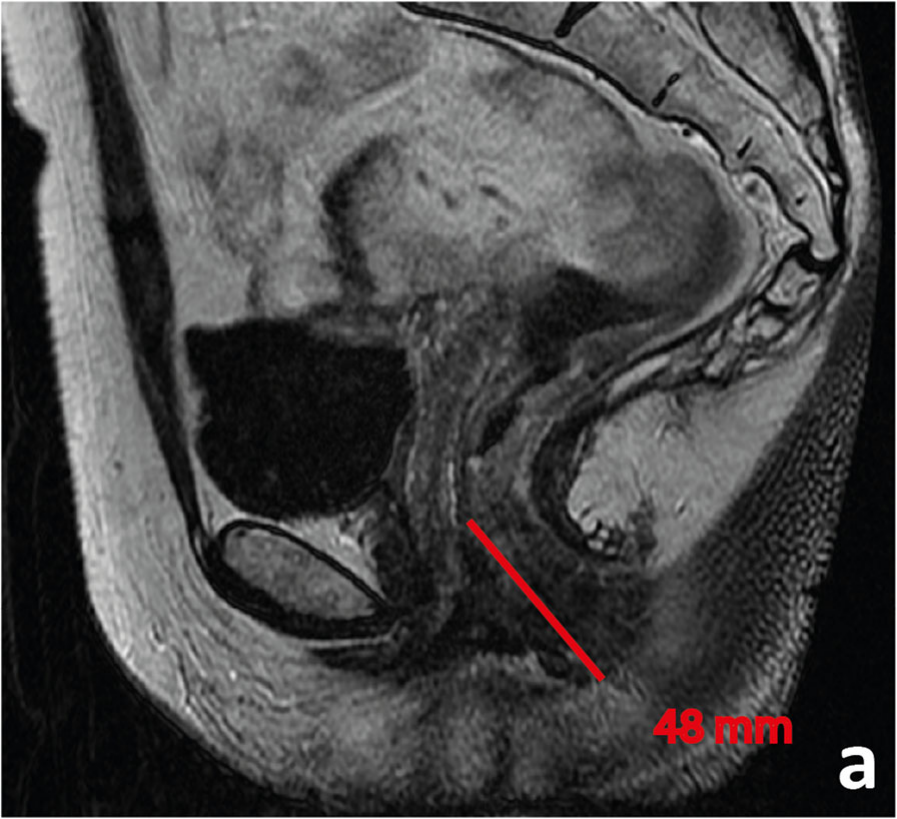
A low rectal cancer is depicted with its lower edge 48 mm above the anal verge. Measurement is made from the central axis of the anus to the central axis of the rectum at the plane of the lower edge of the tumour

**Fig. 20:**
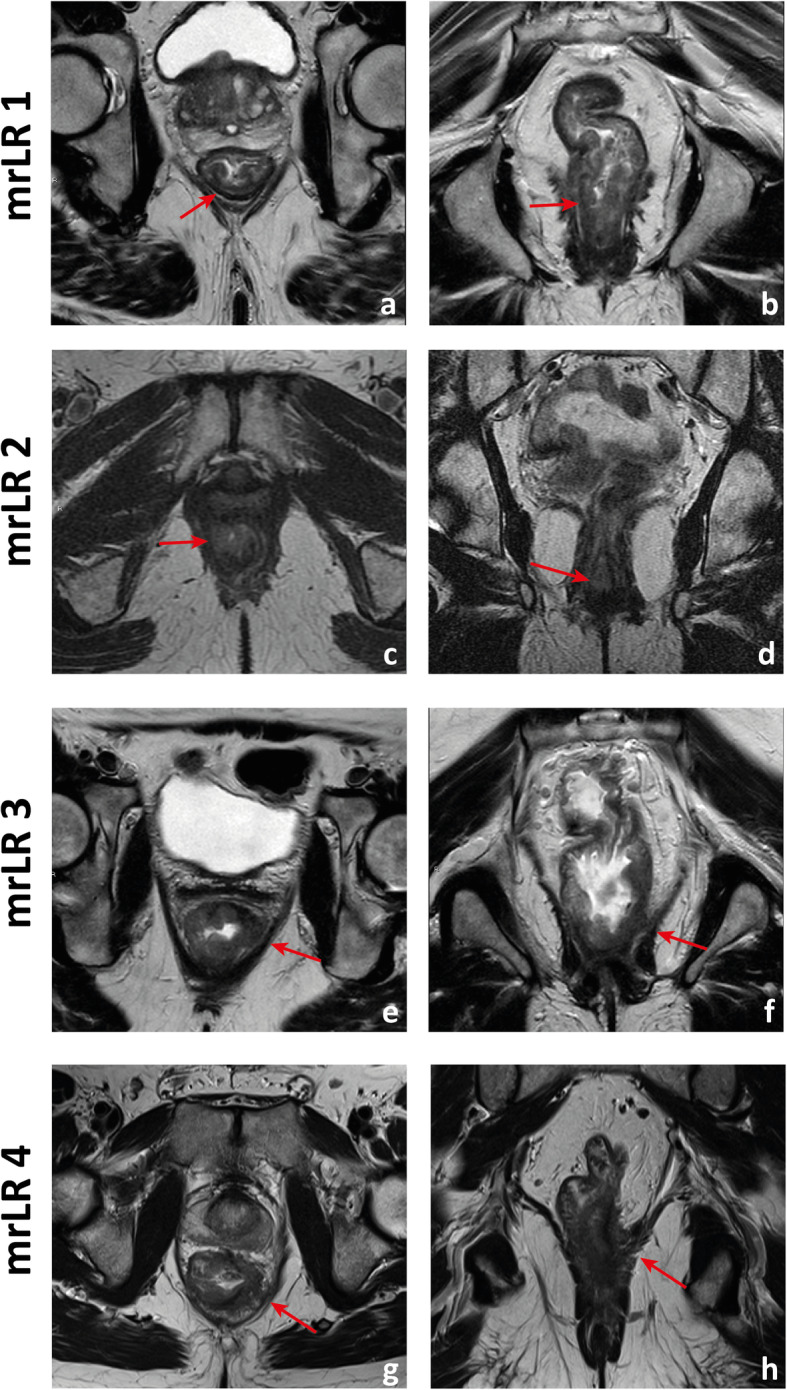
Four low rectal cancers from 4 different patients are depicted. In **a** and **b**, a posterior mrT1sm3/early T2 tumour is depicted. It is classified as LR1 according to the low rectal cancer classification given it does not extend through the full thickness of the rectal wall. In **c** and **d**, a right anterior quadrant mrT2 tumour extending inferiorly into the anal canal is depicted, which invades the full thickness of the internal anal sphincter without extending into the intersphincteric plane—mrLR2. In **e** and **f**, a T3 tumour extending into the low mesorectum to within 1 mm of the left levator muscle is classified as mrLR3; in **g** and **h**, a low tumour extends through the mesorectal fat reaching the left levator muscle, therefore classified as mrLR4

A tumour with its lower edge less than 6 cm above the anal verge is considered a low rectal cancer [63](Fig. 19). Its prognosis is worse, with higher rates of local recurrence and poorer survival [63]. These figures may result from the inferior mesorectal tapering and closer proximity of tumours to the pelvic floor and middle pelvic compartment structures, requiring more mutilating surgical procedures and being associated with higher rates of margin positivity [64]. A specific MR imaging staging system was developed by Shihab et al. to address the particularities of low rectal cancer and aid in surgery planning [63, 65, 66]. According to it, tumours confined to the bowel wall not extending through its full thickness are considered mrLR1, tumours replacing the muscle coat but not reaching the intersphinteric plane are considered mrLR2, tumours invading the intersphincteric plane or lying within 1 mm to the levator muscle are considered mrLR3 and tumours invading the external anal sphincter and being within 1 mm and beyond the levator muscle, with or without invading adjacent structures, are considered mrLR4 (Fig. 20) [66]. mrLR1 tumours confined to the low rectum may be approached by LAR, in which the mesorectum is removed en bloc down to the pelvic floor. mrLR1 tumours extending into the anal canal may be approached with a LAR + intersphincteric dissection [4]. Low colorectal anastomosis or coloanal anastomosis may be performed upon favourable sphincter competence in these procedures, to avoid a permanent stoma, but the rate of anastomotic leak and pelvic sepsis is increased and may significantly reduce patient quality of life. mrLR2 may be approached with standard abdominoperineal excision (SAPE), also known as an intersphincteric APE, in which the distal colon, rectum and anal sphincter are removed en bloc [4, 67]. When surgical treatment is considered for mrLR3-4, an extralevator abdominoperineal excision (ELAPE) is oncologically safer (less chance of R1 resection), in which dissection along the mesorectal fascia ends caudally at the levator origin and is met by a perineal dissection along the outer surface of the levators [4, 68]. ELAPE is associated with lower perforation and margin involvement rates, and consequent lower local recurrence rates than SAPE, albeit at the cost of higher perineal morbidity [68]. For tumours located both above and below the puborectalis sling or located anteriorly, at the level of the prostate, pelvic exenteration may be more appropriate [69]. In the particular case of very low rectal tumours (< 4 cm from the anal verge), mrT > 2 may by itself be an indication for neoadjuvant therapy according to European guidelines, whereas for higher locations, it may be reserved for mr > T3b if good quality TME can be assured [33]. Examples of mrLR1 - 4 are given in Fig. 20.

##### Keep in mind

In low rectal tumours, additional oblique axial and coronal T2-WI planes perpendicular and parallel to the long axis of the anal canal, respectively, should be acquired when needed. The anal verge may be identified on sagittal T2-WI as the lower limit of the perianal hypointense skin change (Fig. 19).

#### The mucinous rectal cancers

**Fig. 21:**
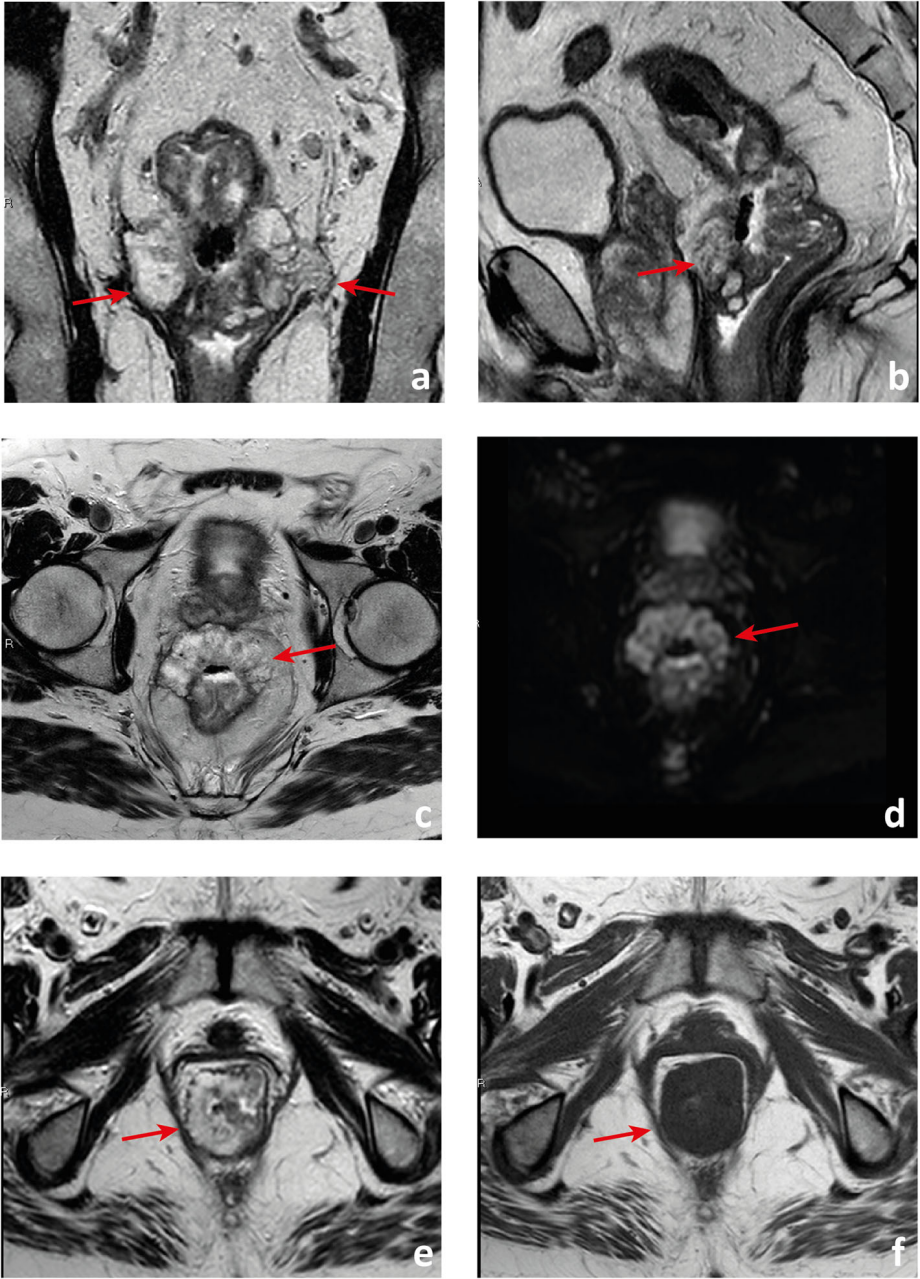
In **a** to **c**, we observe a low/mid rectal cancer with a predominance of high signal intensity in accordance with a mucinous histologic subtype. It presents with bilateral (arrows in **a**) and anterior (arrow in **b**) transgression of the mesorectal fascia, extending into the pelvic sidewall and middle pelvic compartment, respectively. It may not be easy to define its exact boundaries due to the approximation of signal intensity to that of fat (arrow in **c**). b0 DWI images are fat-suppressed T2-WI and may aid in the distinction (arrow in **d**). T1WI may work even better, namely to define or exclude a fat plane between tumour and circumferential margin of resection. In **e**, a low mucinous rectal cancer appears to invade the levator muscle on T2-WI but in the corresponding T1WI (**f**), we see a thin fat plane between the two.

Mucinous adenocarcinomas comprise 10–20% of all rectal cancers, tend to affect younger patients and are defined histologically by the presence of extracellular mucin in more than 50% of the tumour stroma [69]. There is an association with a history of inflammatory bowel disease and pelvic radiotherapy [69]. This histologic subtype may be associated with a higher T stage upon diagnosis, a greater risk of metachronous metastases and a worse response to chemoradiation, but results regarding the impact on overall survival are conflicting [70, 71]. Although the definition is histologic, it may be that mucinous tumours are more efficiently detected on T2-WI MR imaging than on endoscopic biopsy, imaging definition being more than 50% high signal intensity areas within the tumour—areas with a signal intensity similar to or brighter than that of the mesorectal fat [69, 71]. MR imaging-detected mucinous tumours carry a similarly worse prognosis relative to histology, with poorer responses to chemoradiation and worse disease-free survival [69] (Fig. 21).

##### Keep in mind

Whenever in doubt, DWI b0 may be useful for the distinction between fat signal, which will be suppressed, and mucin, which will remain bright. Also, we find high-resolution T1-WI may help define the boundary between mucin and fat (Fig. 21).

#### Rectal cancer staging templates

**Fig. 22:**
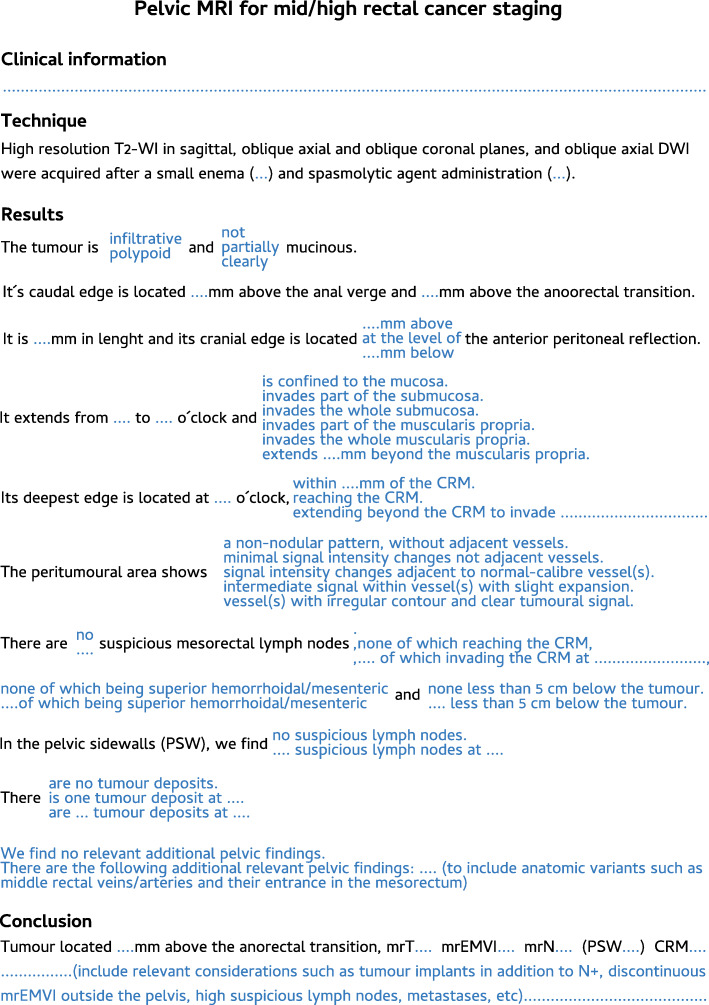
A standardised report proforma for mid/high rectal cancer staging is depicted. In blue, a single option should be chosen per field

**Fig. 23:**
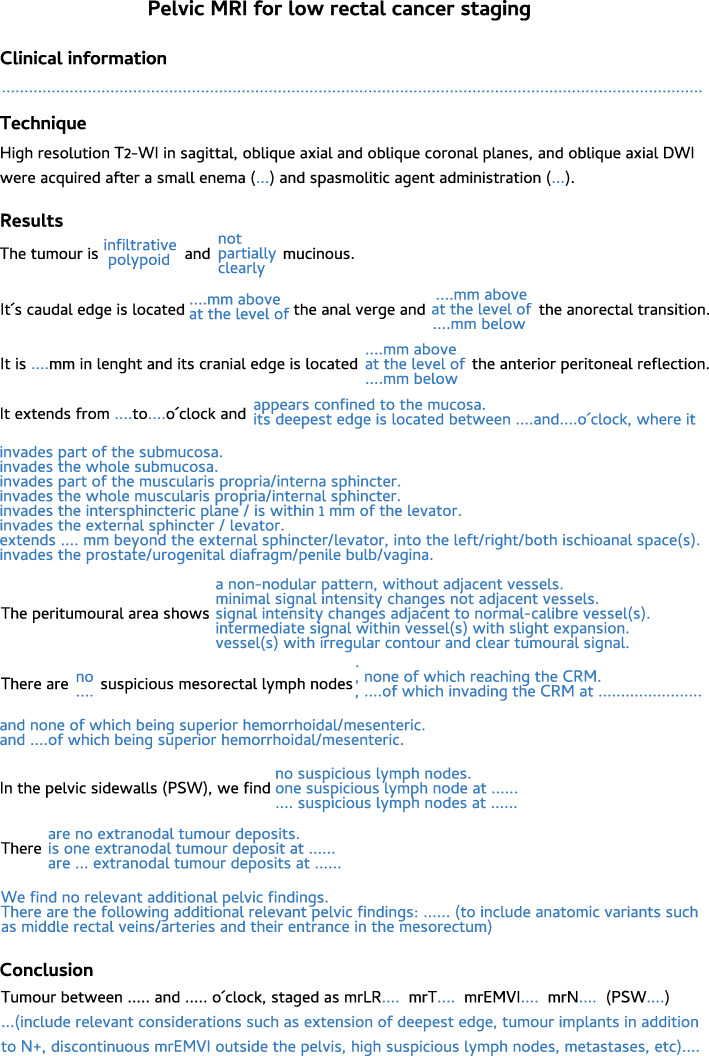
A standardised report proforma for low rectal cancer staging is depicted. In blue, a single option should be chosen per field

Figures 22 and 23 depict our suggested templates for the staging of mid/high and low rectal cancers respectively. In this section, we discuss the small deviations from SAR and ESGAR consensus guidelines and their reasoning.

Our patients routinely perform a small enema shortly before MR imaging to empty the rectum, which significantly reduces susceptibility artefacts due to air content, improvement being particularly relevant for DWI [72]. Intravenous butilscopolamine is also administered routinely at our centre in the absence of contraindications, between scout and (oblique axial) T2-WI acquisitions, to minimise artefacts due to peristalsis. Neither the use of small enema nor the use of spasmolytic agents has reached consensus by SAR or ESGAR expert panels and is therefore considered optional according to their respective guidelines [13, 73].

Our acquisition protocol is exhibited in Table 1. We routinely perform DWI in staging examinations to help locate small lesions and to serve as a baseline for response assessment. Although DWI is a recommended sequence according to SAR consensus guidelines, it is considered optional upon staging according to ESGAR consensus guidelines [73, 74]. We do not administer intravenous contrast for rectal cancer staging, which is optional according to both guidelines. The exception is a suspicion of pelvic sepsis or fistulization, in which case we find contrast very helpful to delineate abscesses and fistulous tracts respectively. We include an unenhanced T1 sequence, as recommended by SAR [13, 73], which provides an overview of the whole pelvis and may help characterize incidental bone ou genito-urinary lesions. It may also help delimit mucinous tumours, as discussed in the “The mucinous rectal cancers” section.

With respect to staging key points, all of our patients are staged with MR imaging unless an absolute contraindication is known, even early tumours, and as such, we have decided to include early tumour sub-staging in our templates as by Balyasnikova et al. [62], which is not part of either SAR or ESGAR consensus guidelines [13, 73]. Also, we have kept the mrLR classification for low rectal tumours [66] given it is well known by our multidisciplinary team. Even though the specific mrLR term is not utilised in the templates by SAR or ESGAR, the depth of invasion subclass options for low tumours is presented similarly in both [13, 73]. We have used the 5-point scale by Smith et al. [43] for mrEMVI assessment since the very beginning of our practice, and we find it helpful for less experienced radiologists. As such, we kept it, whereas SAR and ESGAR have adopted a simpler 3-point and 2-point scale for that purpose, respectively [13, 73]. Finally, our templates leave node involvement interpretation open, whereas both SAR and ESGAR reporting templates adopt strict mixed size-morphology criteria [13, 73]. The reason for this deviation is our own node-by-node analysis experience based on such criteria, which we found suboptimal [74]. We rely on morphologic criteria irrespective of size, namely shape, contour, heterogeneity and chemical shift effect, as explained in the “Lymph node involvement and tumour deposits” section.

## Conclusions

Pelvic MR imaging is the pillar of clinical rectal cancer staging and the basis for optimal multidisciplinary patient management decision-making. A thorough and systematic knowledge of the relevant normal anatomy and variants, of the key staging imaging features and of the particularities of early, low and mucinous tumours is mandatory for rectal cancer staging MR image interpretation.

## Data Availability

Not applicable.

## Abbreviations

AJCC: American Joint Committee on Cancer
CRM: Circumferential margin of resection
DWI: Diffusion-weighted imaging
EMVI: Extramural venous invasion
ENTD: Extranodal tumour deposit
ESMO: European Society of Medical Oncology
MR: Magnetic resonance
NCCN: National comprehensive cancer network
OR: Odds ratio
T2-WI: T2-weighted imaging
TEM: Transanal endoscopic microsurgery
TME: Total mesorectal excision
TNM: Tumour node metastases staging system
